# *SLC6A14* Is a Genetic Modifier of Cystic Fibrosis That Regulates *Pseudomonas aeruginosa* Attachment to Human Bronchial Epithelial Cells

**DOI:** 10.1128/mBio.02073-17

**Published:** 2017-12-19

**Authors:** Michelle Di Paola, Amber J. Park, Saumel Ahmadi, Elyse J. Roach, Yu-Sheng Wu, Michaela Struder-Kypke, Joseph S. Lam, Christine E. Bear, Cezar M. Khursigara

**Affiliations:** a Programme of Molecular Medicine, Hospital for Sick Children, Toronto, Ontario, Canada; b Department of Biochemistry, University of Toronto, Toronto, Ontario, Canada; c Department of Molecular and Cellular Biology, University of Guelph, Guelph, Ontario, Canada; d Department of Physiology, University of Toronto, Toronto, Ontario, Canada; e Molecular and Cellular Imaging Facility, Advanced Analysis Centre, University of Guelph, Guelph, Ontario, Canada; Georgia Institute of Technology

**Keywords:** *CFTR* mutation, l-arginine uptake, *Pseudomonas aeruginosa*, SLC6A14, airway epithelia, bacterial colonization, cystic fibrosis, modifier gene

## Abstract

Cystic fibrosis (CF) is caused by mutations in the *CFTR* gene and is associated with progressive and ultimately fatal infectious lung disease. There can be considerable variability in disease severity among individuals with the same *CFTR* mutations, and recent genome-wide association studies have identified secondary genetic factors that contribute to this. One of these modifier genes is *SLC6A14*, which encodes an amino acid transporter. Importantly, variants of this gene have been associated with age at first acquisition of *Pseudomonas aeruginosa*. In this study, we aimed to determine the function of SLC6A14 in airway epithelia and how it might affect colonization by *P. aeruginosa*. We show that *SLC6A14* is expressed in respiratory epithelial cells and transports l-arginine out of the airway surface liquid (ASL). Exposure of airway epithelia to flagellin from *P. aeruginosa* led to upregulation of *SLC6A14* expression and increased SLC6A14-dependent uptake of l-arginine from the ASL. In support of the hypothesis that l-arginine affects *P. aeruginosa* attachment, we showed that l-arginine supplementation promoted *P. aeruginosa* attachment to an abiotic surface in a dose-dependent manner. In a coculture model, we found that inhibition of SLC6A14-dependent l-arginine transport enhanced *P. aeruginosa* attachment. In *Slc6a14*^−/y^ (knockout) mice, *P. aeruginosa* attachment to lung tissue was also significantly enhanced. Together, these findings suggest that SLC6A14 activity plays a role in the modification of the initial stages of airway infection by altering the level of l-arginine in the ASL, which in turn affects the attachment of *P. aeruginosa*.

## IMPORTANCE

CF patients with shared *CFTR* gene mutations show significant variability in their clinical presentation of infectious lung disease. Genome-wide association studies have been used to identify secondary genetic factors that may explain the variable susceptibility to infection by opportunistic pathogens, including *P. aeruginosa*, the leading cause of pathogen-induced lung damage in nonpediatric CF patients. Once identified and characterized, these secondary genetic modifiers may allow for the development of personalized medicine for patients and ultimately the extension of life. In this study, we interrogated the biological role of one of these modifiers, *SLC6A14*, and showed that it contributes to host defense by depleting extracellular arginine (an attachment-promoting metabolite for *P. aeruginosa*) from the airway surface liquid.

## INTRODUCTION

Over 25 years has passed since the identification of the gene that is responsible for the heritable multiorgan disorder known as cystic fibrosis (CF) (1–3). While multiple mutations in the CF transmembrane conductance regulator (*CFTR*) gene have been identified, >70% of CF patients carry the F508del mutation (3), which results in decreased surface expression of the CFTR protein. Defects in CFTR affect ion transport across the apical surface of epithelia in multiple tissue types, most notably the lungs and gastrointestinal tract, resulting in the production of dehydrated and viscous surface fluids. The physiological changes that occur in the lung prevent normal mucociliary clearance of foreign debris and predispose patients to chronic infectious lung disease (4). Recurrent and worsening infections by a variety of opportunistic pathogens lead to progressive declines in pulmonary function that require lung transplantation or result in patient death. Importantly, while the contribution of *CFTR* to CF-associated lung disease has been well established (5, 6), it has been noted that patients with the same *CFTR* genotype often present with high degrees of phenotypic variation. Accordingly, large-scale genome-wide association studies (GWAS) have recently been completed in an attempt to identify secondary modifiers of CF-related morbidities (7–9). One of the secondary modifiers identified, *SLC6A14*, is associated with advanced early disease, including intestinal blockage at birth (meconium ileus), pediatric lung disease, and age at first *Pseudomonas aeruginosa* infection (10, 11). Although it is known that the *SLC6A14* gene encodes an amino acid transporter, proposed to mediate the uptake of neutral and cationic amino acids at the apical surface of multiple types of epithelia, it is unclear how the gene product influences the expression of disease. Earlier studies by Galietta and colleagues that identified an electrogenic dibasic amino acid transporter on the apical membrane of primary bronchial epithelial cells hypothesized that this transporter could be important in maintaining a low amino acid concentration in the airway surface liquid (ASL) (12). This hypothesis is reinforced by the observation that the availability of key amino acids, especially arginine, can influence the behavior of *P. aeruginosa* within CF lung-like environments (13, 14). In this study, we investigated the specific role of SLC6A14 in l-arginine transport at the apical membrane of airway epithelia in the context of airway infection. We aimed to determine if reduced function of SLC6A14 might, by increasing the l-arginine levels in the ASL, result in increased attachment of *P. aeruginosa* to airway epithelia. This would provide a mechanism through which the *SLC6A14* gene modifies *P. aeruginosa* lung infection in CF patients.

## RESULTS

### *Slc6a14* and *SLC6A14* expression confers l-arginine transporter activity on the apical membrane of murine tracheal and human airway epithelia, respectively.

**FIG 1:**
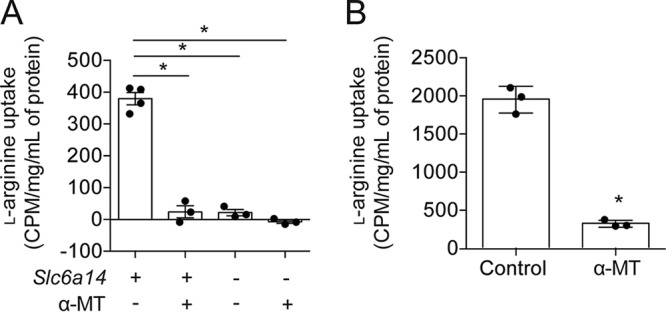
SLC6A14 is an important modulator of l-arginine uptake in airway epithelium. l-[2,3-^3^H]arginine was used to monitor the intracellular uptake of arginine from the apical surfaces of airway epithelium harvested from mice or cultured with a human cell line (Calu-3). (A) l-[2,3-^3^H]arginine uptake, expressed in counts per minute per concentration of whole tissue lysate, significantly decreased following genetic knockout of *Slc6a14* (*Slc6a14*^−/y^) or SLC6A14 inhibition with α-MT in wild-type (*Slc6a14*^+/y^) mice. Data points represent independent biological replicates ± the standard deviation. Asterisks indicate a *P* value of <0.05 (one-way analysis of variance with Tukey’s multiple-comparison test). (B) Significant reduction in l-[2,3-^3^H]arginine uptake upon inhibition of SLC6A14 with α-MT in Calu-3 airway epithelial monolayers. Data points represent three independent biological replicates (each consisting of four technical replicates) ± the standard deviation. Asterisks indicate a *P* value of <0.05 (two-tailed unpaired *t* test).

Disruption of *Slc6a14* gene expression in mice does not lead to gross phenotypic changes or changes in survival (15). However, we hypothesized that SLC6A14 could play an important role in the regulation of l-arginine levels at the surface of bronchial cells, which in turn could affect certain pathogenic processes. To show that SLC6A14 plays a primary role in the transport of amino acids across the apical membrane of the murine tracheal epithelium, we compared the uptake of l-[2,3-^3^H]arginine by performing *ex vivo* studies of both *Slc6a14*^+/y^ (wild-type) and *Slc6a14*^−/y^ (knockout) mice. Real-time PCR (RT-PCR) data verified the knockout of *Slc6a14* in the murine tissues (Fig. S1A). The uptake of l-[2,3-^3^H]arginine from the luminal fluid contents of a closed tracheal segment from *Slc6a14*^−/y^ mice was significantly lower than that of their wild-type littermates (Fig. 1A). In addition to these findings, we confirmed previous studies showing that the arginine transport function mediated by SLC6A14 is inhibited by α-methyl-dl-tryptophan (α-MT) (16, 17). Injection of α-MT into the luminal contents of closed tracheal segments inhibited l-[2,3-^3^H]arginine uptake in *Slc6a14*^+/y^ mice to the same low levels observed in closed tracheal segments from *Slc6a14*^−/y^ mice (Fig. 1A). Further, administration of α-MT failed to exert an inhibitory effect on l-[2,3-^3^H]arginine uptake in tracheal segments from *Slc6a14*^−/y^ mice (Fig. 1A). Together, these murine studies imply a primary role for SLC6A14 in l-arginine removal from the ASL.

10.1128/mBio.02073-17.1 FIG S1  (A) Quantitative RT-PCR analysis was used to confirm the knockout of transporter expression in *Slc6a14*^−/y^ (knockout) mice relative to that in the wild-type control strain. (B) *SLC6A14* is expressed in Calu-3 lung epithelial cells. (C) *SLC6A14* is expressed in HBE (upper) and CFBE (lower) primary airway cell cultures. The asterisk indicates a *P* value of <0.05 (two-tailed unpaired *t* test). The values to the left of the blots in panels B and C are molecular sizes in kilobase pairs. Download FIG S1, TIF file, 12 MB. Copyright © 2017 Di Paola et al. 2017 Di Paola et al. This content is distributed under the terms of the Creative Commons Attribution 4.0 International license.

Next, we examined the role of SLC6A14 in the mediation of l-arginine uptake across the apical membrane of human bronchial epithelium. First, we showed that *SLC6A14* is expressed in Calu-3 cells, a human cell line that has been used to model the airway epithelial barrier (18) (Fig. S1B). We then quantified the activity of SLC6A14 in the Calu-3 cell line by using an approach similar to that used in the aforementioned murine studies. Specifically, l-[2,3-^3^H]arginine uptake from the fluid bathing the apical membrane of a confluent monolayer of Calu-3 cells was measured and the effect of α-MT was determined. Indeed, l-arginine uptake by Calu-3 cells was inhibited by α-MT (Fig. 1B), supporting the hypothesis that SLC6A14 mediates this function in this cell line.

### *SLC6A14* expression in human airway epithelial cultures is responsive to purified FLA-PA.

**FIG 2:**
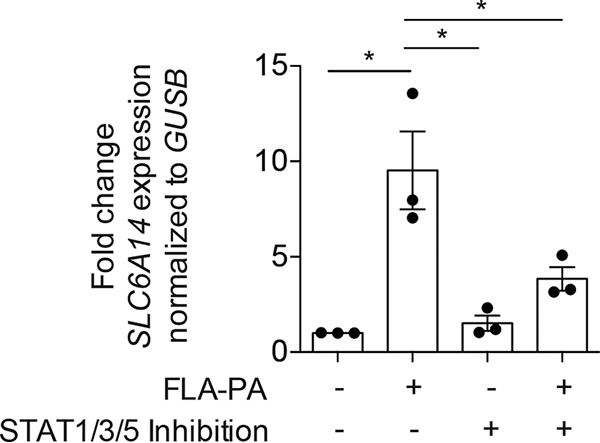
Expression of *SLC6A14* in a human epithelial cell line is increased by flagellin in a STAT-dependent manner. *SLC6A14* expression in Calu-3 monolayers was measured after a 2-h exposure to FLA-PA with or without simultaneous exposure to the STAT1/3/5 combination inhibitor nifuroxazide. Data points represent three independent biological replicates (each consisting of three technical replicates) ± the standard deviation. Asterisks indicate a *P* value of <0.05 (one-way analysis of variance with Tukey’s multiple-comparison test).

As GWAS have shown that polymorphisms in SLC6A14 associate with age at first *P. aeruginosa* infection in CF patients, we tested the hypothesis that *SLC6A14* expression would be responsive to pathogen-host interactions at the apical surface of the airway epithelium. Addition of purified flagellin from *P. aeruginosa* (FLA-PA) to the ASL bathing confluent Calu-3 monolayers was shown to enhance the expression of *SLC6A14* >9.5-fold at 2 h (Fig. 2) and 24.6-fold at 6 h (Fig. S2). For comparison, we also examined the effects of treatment with purified lipopolysaccharides (LPS) derived from *P. aeruginosa* on the expression of *SLC6A14*. Treatment with LPS showed a minor enhancement of *SLC6A14* expression of 1.7-fold at 6 h (Fig. S3). Next, we used the HaploReg v2.0 database to predict potential protein binding sites on the *SLC6A14* gene. This analysis showed that the *SLC6A14* gene promoter bears predicted binding sites for signal transducer and activator of transcription (STAT) proteins. Specifically, of several variants that are associated with CF lung disease, RS2208263 and RS4824377 fall in a region where there is evidence of both STAT1 and STAT3 transcription factor binding. Accordingly, the application of a pan-inhibitor of STAT1, -3, and -5 (nifuroxazide) was effective in ameliorating the effect of flagellin treatment (Fig. 2), thus supporting the role of signaling through one or more of these factors.

10.1128/mBio.02073-17.2 FIG S2  Expression of *SLC6A14* can be modulated by FLA-PA. Following a 6-h exposure to FLA-PA, the *SLC6A14* mRNA level in Calu-3 cells was higher than that in untreated controls. The asterisk indicates a *P* value of <0.05 (two-tailed unpaired t test). Download FIG S2, TIF file, 1.7 MB. Copyright © 2017 Di Paola et al. 2017 Di Paola et al. This content is distributed under the terms of the Creative Commons Attribution 4.0 International license.

10.1128/mBio.02073-17.3 FIG S3  Expression of *SLC6A14* can be modulated by LPS derived from *P. aeruginosa*. Following a 6-h exposure to LPS from *P. aeruginosa*, *SLC6A14* expression in Calu-3 cells was higher than that in untreated controls. The asterisk indicates a *P* value of <0.05 (two-tailed unpaired t test). Download FIG S3, TIF file, 0.1 MB. Copyright © 2017 Di Paola et al. 2017 Di Paola et al. This content is distributed under the terms of the Creative Commons Attribution 4.0 International license.

**FIG 3:**
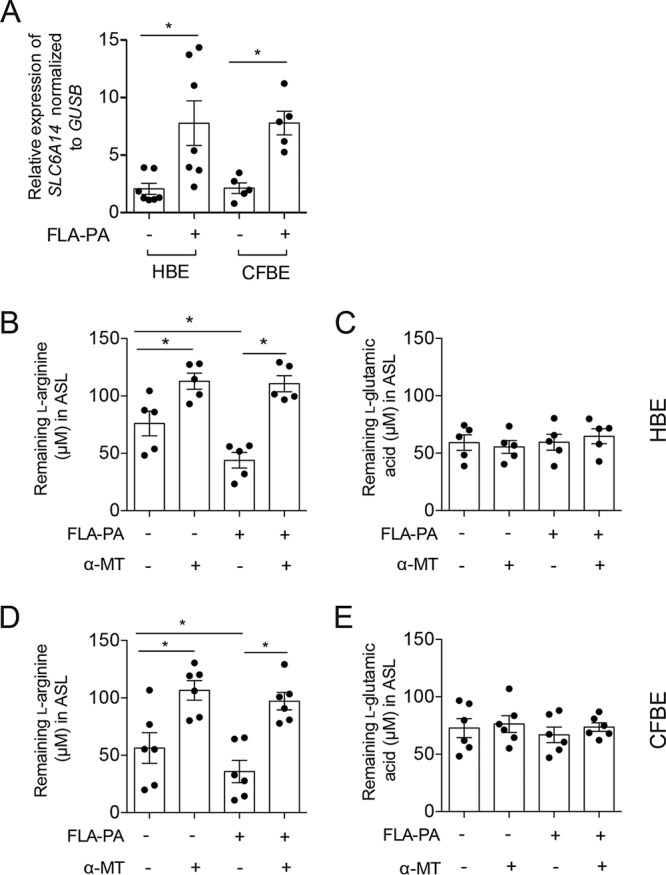
Expression of *SLC6A14* and function of SLC6A14 in primary HBE cells can be modulated by FLA-PA. (A) Following a 6-h exposure to FLA-PA, the level of *SLC6A14* mRNA in primary epithelial cells was higher than that in untreated controls. Importantly, the measured increase in *SLC6A14* expression corresponded to reduced levels of l-arginine remaining in the ASL of primary HBE (B) and CFBE (D) cell cultures, indicating higher levels of cellular uptake. This effect was blocked by SLC6A14 inhibition by α-MT treatment. In contrast, cellular uptake of a SLC6A14 nonsubstrate, l-glutamic acid, was not affected in HBE (C) or CFBE (E) cells with modulated levels of *SLC6A14* expression or in tissues treated with α-MT. Data points for gene expression (A) represent independent biological replicates (each consisting of three technical replicates). All other data points represent biological replicates ± the standard deviation. Asterisks indicate a *P* value of <0.05 (one-way analysis of variance with Tukey’s multiple-comparison test).

Similar experiments were conducted with primary human bronchial epithelial (HBE) cells derived from healthy donors or patients with CF (CFBE cells). We observed that *SLC6A14* was also expressed in primary human bronchial cultures grown at the air-liquid interface by using Transwell permeable supports (Fig. S1C). As seen with the Calu-3 cells, when FLA-PA was added to the ASL of primary HBE and CFBE cell cultures, a significant increase in the expression of *SLC6A14* was observed (Fig. 3A).

To determine if the FLA-PA-induced increases in *SLC6A14* expression had functional consequences in HBE and CFBE cells, we quantified amino acid concentrations in the ASL by high-performance liquid chromatography (HPLC) (Fig. 3B to E; Fig. S4A and B). Measurements were taken 1 h after the ASL was spiked with l-arginine or l-glutamic acid (a non-SLC6A14 substrate control). Consistent with the increased quantities of *SLC6A14* mRNA (Fig. 3A), we found that the concentration of spiked l-arginine remaining in the ASL of HBE (Fig. 3B) and CFBE (Fig. 3D) cell cultures at 1 h was significantly lower with exposure to FLA-PA than without it. In contrast, we did not observe changes in the concentration of the control amino acid (l-glutamic acid) in the ASL from primary cultures following stimulation with FLA-PA (Fig. 3C and E), pointing to the specificity of this effect for SLC6A14 substrates. The sensitivity of this effect to SLC6A14 inhibition was also demonstrated by α-MT treatment. Specifically, significantly larger quantities of the spiked l-arginine remained in the ASL of cultures treated with α-MT than in the ASL of cultures not treated with α-MT (Fig. 3B and D). The l-glutamic acid concentrations in ASL were not altered by α-MT treatment (Fig. 3C and E), further supporting the specificity of the assay for SLC6A14 function. Together, these findings show that *SLC6A14* expression is responsive to the presence of immunogenic *P. aeruginosa* cell surface structures and that this response occurs irrespective of the *CFTR* genotype (Table S2).

10.1128/mBio.02073-17.4 FIG S4A  HPLC measurements of amino acids in ASL. Selected chromatographic traces from the ASL of HBE airway cell cultures. Traces were acquired from samples treated with either amino acids only (l-arginine and l-glutamic acid) or amino acids in combination with either FLA-PA or α-MT. Peaks representing l-arginine and l-glutamic acid from each condition are indicated (arrows). A larger peak for l-arginine, indicating an overall higher concentration of arginine, was observed following inhibition of SLC6A14 with α-MT. Following FLA-PA treatment, smaller peaks were observed, indicating less l-arginine present in the ASL. These trends were not observed for l-glutamic acid peaks. AU, arbitrary units. Download FIG S4A, TIF file, 14 MB. Copyright © 2017 Di Paola et al. 2017 Di Paola et al. This content is distributed under the terms of the Creative Commons Attribution 4.0 International license.

10.1128/mBio.02073-17.5 FIG S4B  Selected chromatographic traces from the ASL of CFBE airway cell cultures. AU, arbitrary units. Download FIG S4B, TIF file, 14 MB. Copyright © 2017 Di Paola et al. 2017 Di Paola et al. This content is distributed under the terms of the Creative Commons Attribution 4.0 International license.

10.1128/mBio.02073-17.9 TABLE S1  Sequences of primers used for quantitative RT-PCR assays. Download TABLE S1, DOCX file, 0.02 MB. Copyright © 2017 Di Paola et al. 2017 Di Paola et al. This content is distributed under the terms of the Creative Commons Attribution 4.0 International license.

10.1128/mBio.02073-17.10 TABLE S2  Genotype table. The *CFTR* genotypes of the primary CFBE cell cultures used for each of the assays are indicated. Download TABLE S2, DOCX file, 0.02 MB. Copyright © 2017 Di Paola et al. 2017 Di Paola et al. This content is distributed under the terms of the Creative Commons Attribution 4.0 International license.

### l-Arginine promotes surface attachment of *P. aeruginosa*.

**FIG 4:**
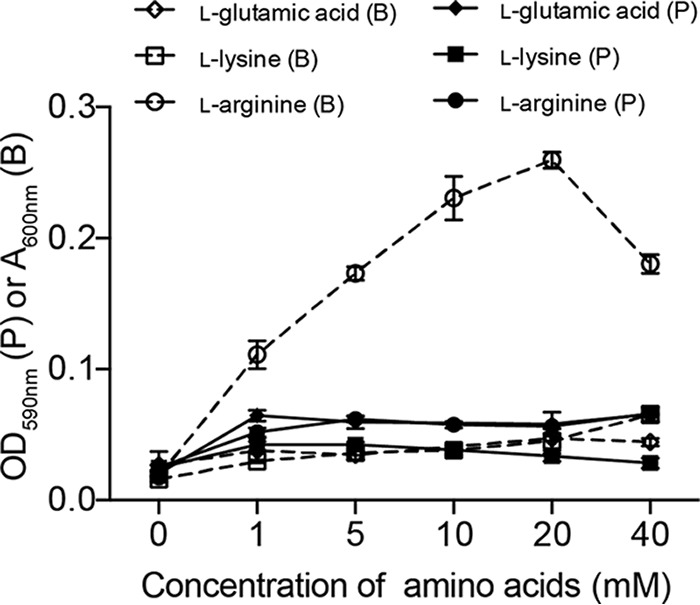
l-Arginine promotes surface-associated monoculture growth of *P. aeruginosa* under epithelial-cell-free conditions. Free-swimming (planktonic) and surface-associated (biofilm) growth of *P. aeruginosa* was assessed in 24-h microtiter biofilm assays by using various concentrations of SLC6A14 substrates (l-arginine and l-lysine) and a nonsubstrate (l-glutamic acid) in HEPES buffer. Data represent the average biofilm biomass (*A*_590_, open symbols, dashed lines) or planktonic cell density (OD_600_, closed symbols, solid lines) of four biological replicates per treatment ± the standard deviation. Abbreviations: P, planktonic; B, biofilm.

Previous studies have identified the amino acids present in CF sputum (13) and demonstrated that some of these compounds can stimulate surface colonization and biofilm formation in *P. aeruginosa* (14). In this study, we confirmed that the SLC6A14 transporter substrate l-arginine stimulated the attachment of *P. aeruginosa* to an abiotic surface in a dose-dependent manner (Fig. 4). In contrast, l-arginine supplementation did not have an appreciable effect on the growth of planktonic bacteria. Other amino acids, including l-lysine (SLC6A14 substrate) and l-glutamic acid (nonsubstrate), showed a negligible effect compared to that of l-arginine on the levels of surface attachment and the associated biofilm biomass formed by *P. aeruginosa*.

### Functional SLC6A14 is required to mitigate attachment of *P. aeruginosa* to primary bronchial epithelial cells.

**FIG 5:**
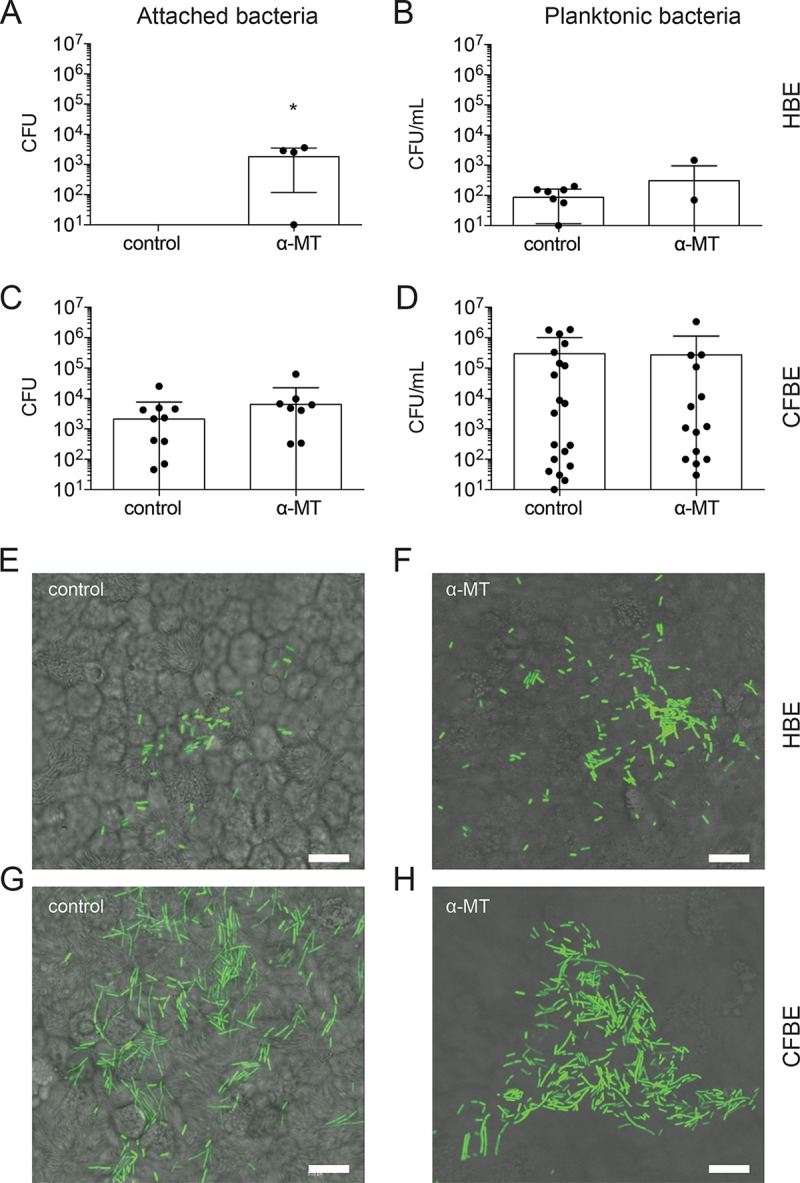
Inhibition of SLC6A14 results in increased bacterial attachment to HBE monolayers. Quantification of bacterial CFU attached to the epithelial cell monolayer or collected from the infection medium (planktonic). (A) Treatment of HBE cell monolayers with α-MT results in a significant increase in the amounts of attached *P. aeruginosa* recovered after 8 h of infection (*P* < 0.05). (B) Viable bacterial counts in infection medium show that treatment with α-MT does not have a significant effect on planktonic growth of *P. aeruginosa* in HBE cell cocultures. (C and D) Treatment with α-MT did not influence attached or planktonic bacterial cell counts in CFBE cocultures. Individual data points are shown for biological replicates with CFU counts above the lower limit of detection ± the standard deviation. Numbers of patient samples with CFU counts below the detection level: A, 9 and 1; B, 2 and 3; C, 11 and 7; D, 1 and 1 (control and treated, respectively). Asterisks indicate a *P* value of <0.05 (two-tailed unpaired *t* test assuming equal standard deviation [B and D], not assuming normal distribution [A, Mann-Whitney test], or not assuming equal standard deviation [C, Welch’s correction]). (E to H) Epithelial cell monolayers were imaged by confocal microscopy at 8 h postinfection. Higher numbers of GFP-expressing *P. aeruginosa* bacteria can be seen on the surface of HBE cells treated with α-MT than on that of untreated control HBE cells. CFBE cell monolayers show a greater bacterial burden than HBE cells; however, α-MT did not have an appreciable effect on the quantity of bacteria visualized on the surface of CFBE cell monolayers. Scale bars, 10 μm.

After verifying that α-MT did not affect *P. aeruginosa* growth or biofilm formation directly (Fig. S5), we tested the effect of SLC6A14 inhibition on the growth and attachment of *P. aeruginosa* in the presence of primary HBE and CFBE cultures grown at the air-liquid interface by using Transwell permeable supports (Fig. 5). As expected, the amount of viable bacteria recovered from both the ASL (planktonic bacteria) and epithelial cell monolayer (attached bacteria) of the cocultures was greater in untreated CFBE cultures than in untreated HBE cell cultures. Of the nine patient samples tested, none of the untreated HBE samples showed detectable bacterial counts, indicating low (i.e., below detection) or no attachment to this sample type. This finding provides evidence that supports the validity of the coculture systems, as it is consistent with the increased vulnerability of CF patients to bacterial infection. Importantly, treatment with α-MT significantly increased the attachment of bacteria to HBE cell monolayers (Fig. 5A), suggesting that SLC6A14-dependent changes at the epithelial layer surface were favorable to the surface-associated growth of *P. aeruginosa*. In contrast, treatment with α-MT affected neither bacterial attachment to primary CFBE cells (Fig. 5C) nor planktonic growth of bacteria in the ASL of either cell type (Fig. 5B and D). This pattern was also seen when epithelial-cell-conditioned medium was removed from the cell culture system prior to inoculation with bacteria. Specifically, epithelial-cell-conditioned medium from α-MT-treated HBE cells significantly enhanced monoculture biofilm formation on an abiotic surface (versus untreated HBE cell-conditioned medium), whereas biofilm formation in conditioned medium from CFBE cells treated with α-MT did not differ from that of untreated CFBE cell-conditioned medium (Fig. S6). Bacterial quantification data from the coculture samples were complemented with confocal microscopy of the epithelial cell monolayers at the endpoint (i.e., 8 h of coculture). Higher numbers of green fluorescent protein (GFP)-expressing *P. aeruginosa* bacteria can be observed on the surface of HBE cells treated with α-MT than on that of untreated HBE cells (Fig. 5E to H). Importantly, CFBE monolayers show a greater bacterial burden than HBE cells; however, α-MT did not have an appreciable effect on the quantity of bacteria visualized on the surface of CFBE cell monolayers. For quality control, monolayer integrity was monitored throughout the 8 h of infection of the primary cell coculture studies by recording the transepithelial resistance (TER; minimum threshold, 500 Ω) and by visual inspection of the monolayer (Fig. S7). Monolayers that showed excessive damage at any point during infection were excluded from the study.

10.1128/mBio.02073-17.6 FIG S5  Incubation with α-MT does not affect planktonic growth or surface attachment of *P. aeruginosa* grown in epithelial-cell-free cultures. Microtiter assays were used to assess off-target effects of α-MT on *P. aeruginosa* grown in HEPES buffer. Eight-hour monoculture planktonic growth and surface attachment were not affected by α-MT. Data points represent biological replicates from two independent experiments ± the standard deviation. Separate unpaired *t* tests were performed for each growth mode with or without α-MT. Download FIG S5, TIF file, 1.7 MB. Copyright © 2017 Di Paola et al. 2017 Di Paola et al. This content is distributed under the terms of the Creative Commons Attribution 4.0 International license.

10.1128/mBio.02073-17.7 FIG S6  Conditioned medium from α-MT-treated HBE cells promotes biofilm formation in the absence of epithelial cells. Sterile medium was collected from the apical side of primary epithelial cells following 8 h of incubation with or without α-MT. Ninety-six-well microtiter assays were then used to determine if factors secreted into the medium could affect the surface-associated growth of *P. aeruginosa* under epithelial-cell-free conditions. Data represent the average biofilm biomass (*A*_590_) normalized to planktonic cell density (OD_600_). Data points represent biological replicates from two independent experiments ± the standard deviation. The asterisk indicates a *P* value of <0.05 (*t* test). An unpaired *t* test was used for replicates where the medium was not exposed to primary cells (i.e., unconditioned medium), and paired *t* tests were used for replicates where the medium was not exposed to primary cells to assess the effect within a single patient source with or without α-MT. Download FIG S6, TIF file, 1.4 MB. Copyright © 2017 Di Paola et al. 2017 Di Paola et al. This content is distributed under the terms of the Creative Commons Attribution 4.0 International license.

10.1128/mBio.02073-17.8 FIG S7  Confirmation of epithelial monolayer integrity during *P. aeruginosa* infection. Epithelial monolayer integrity was monitored throughout the 8 h of exposure to *P. aeruginosa* by using TER measurements and visual inspection of the monolayer at ×10 magnification. (A) TER values for all the samples used in the study preinfection (0 h) and postinfection (8 h). Samples were excluded from the study if the TER value reached <500 Ω at any point during the infection (indicated by the dotted line). (B to E) Representative images of HBE and CFBE cell monolayers at 0 and 8 h with TER values for each of the individual samples. (F) Representative image of a CFBE cell sample that was excluded from the study because of excessive monolayer damage. Arrows indicates examples of small (C) and large (F) monolayer defects caused by *P. aeruginosa* infection. Scale bars, 50 μm. No statistical tests of these data were performed. Download FIG S7, TIF file, 19.6 MB. Copyright © 2017 Di Paola et al. 2017 Di Paola et al. This content is distributed under the terms of the Creative Commons Attribution 4.0 International license.

**FIG 6:**
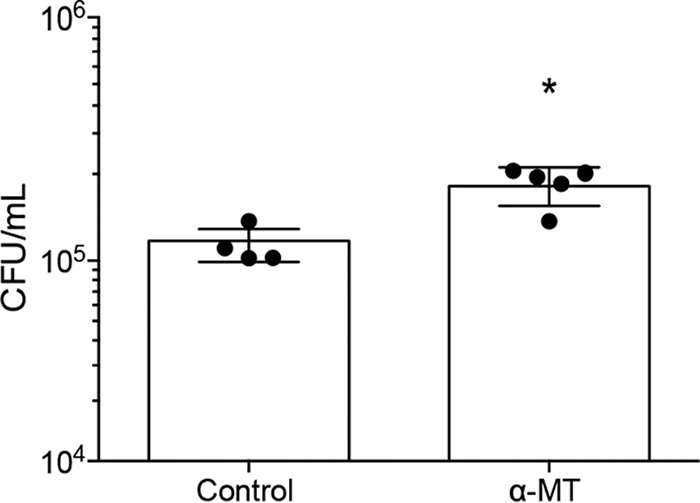
Treatment with α-MT results in increased surface attachment of *P. aeruginosa* to epithelial monolayers generated by an immortalized CFBE cell line. Six-hour coculture assays were used to assess the effects of SLC6A14 inhibition on *P. aeruginosa* attachment to a CFBE cell line (CFBE41o-ΔF508). Data points represent biological replicates ± the standard deviation. The asterisk indicates a *P* value of <0.05 (two-tailed unpaired *t* test).

We reasoned that the effect of α-MT may have been masked by the relatively high basal adherence to the primary CF airway cultures and the considerable heterogeneity in adherence among these cultures. To minimize heterogeneity, we assessed the effect of SLC6A14 inhibition on *P. aeruginosa* adherence to the apical surface of the CFBE41o^−^ (CFBE41o-ΔF508) cell line. We conducted a coculture infection (6 h) of this cell line grown on Transwell filters at the air-liquid interface. Interestingly, *P. aeruginosa* adherence to the surface of these cells was relatively high, but the heterogeneity was minimal among biological replicates (*n* = 4). After inhibition with α-MT (2 mM), we did observe a modest but reproducible increase in *P. aeruginosa* adherence to the surface of CFBE41o^−^ cells (*n* = 5, Fig. 6). These data support our hypothesis that SLC6A14 function normally reduces the initial *P. aeruginosa* adherence in both CF and non-CF airway epithelia.

### An *ex vivo* lung infection model shows greater *P. aeruginosa* attachment to tissues from *Slc6a14*^−/y^ mice.

**FIG 7:**
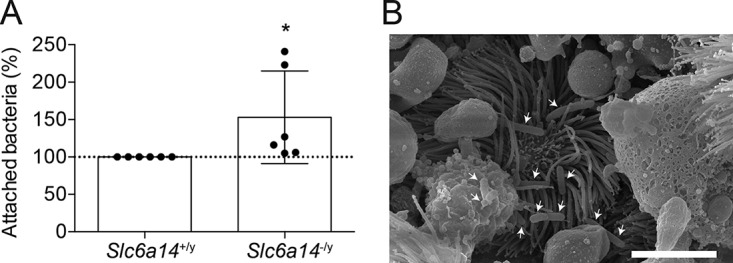
Increased bacterial attachment to lung tissue from *Slc6a14*^−/y^ (knockout) mice. (A) Tissue explants from wild-type and *Slc6a14*^−/y^ mice were exposed to *P. aeruginosa* for 2 h, and bacterial attachment was quantified by counting CFU. Cage- and age-matched pairings indicate significantly greater attachment of bacteria to lung explants from *Slc6a14*^−/y^ mice. Data points represent biological replicates ± the standard deviation. Asterisks indicate a *P* value of 0.0452 (one-tailed paired *t* test). (B) Intimate attachment of *P. aeruginosa* (white arrows) visualized by scanning electron microscopy in tracheal segments exposed to bacteria for 2 h. Scale bar, 5 μm.

Finally, to test the effect of SLC6A14 function on bacterial attachment *ex vivo*, we exposed tissue explants (19) (lungs and trachea) from wild-type (*Slc6a14*^+/y^) and *Slc6a14*^−/y^ (knockout) mice to *P. aeruginosa* for 2 h and proceeded to quantify bacterial attachment. Measurement of attachment to the lungs by standard plating methods showed a significantly higher number of bacteria recovered from *Slc6a14*^−/y^ mouse tissues than from wild-type tissues, indicating that the absence of the arginine transporter in a murine model of pulmonary infection led to an increased bacterial burden in respiratory tract tissues (Fig. 7A). Visual inspection of the tracheal epithelium by scanning electron microscopy indicated that the bacteria were closely associated with the tissue (Fig. 7B).

## DISCUSSION

CF patients display significant variation in lung disease severity, which is conferred by the nature of their *CFTR* mutation, in addition to environmental factors (including exposure to bacterial pathogens) and genetic modifiers. Several genetic modifiers of CF lung disease have been identified (7–11), and investigators in this field are striving to understand their biological role. In the present work, we showed that *SLC6A14*, a genetic modifier of lung disease severity and other CF phenotypes, regulates the availability of arginine at the surface of the lung epithelium by mediating arginine transport out of the ASL. Further, inhibition of SLC6A14 function in primary human respiratory cell cultures or disruption of *Slc6a14* gene expression in mice leads to increased *P. aeruginosa* attachment, a critical initial event in infection. Hence, SLC6A14-mediated arginine uptake in the airway may exert a protective, anti-infective role.

Our data show that the expression of *SLC6A14* and its arginine transport function are enhanced by exposure to immunogenic *P. aeruginosa* surface structures, specifically flagellin, supporting the potential role of this modifier gene in host defense. This concept of a protective role for *SLC6A14* is in good agreement with previous studies showing that early age at first infection with *P. aeruginosa* in CF patients is associated with *SLC6A14* polymorphisms (11) and the observations that *SLC6A14* is upregulated in a cell culture model of cholera infection (20) and in patients with inflammatory bowel disease (21).

While the specific mechanism behind *SLC6A14*’s modification of infectious disease in general, or *P. aeruginosa* attachment specifically, has not been fully elucidated, we tested the hypothesis that a reduction or abrogation of the level of SLC6A14 transporter activity may increase *P. aeruginosa* attachment because of an elevation of the arginine level in the environment of the bacteria. Earlier work has alluded to this concept, specifically, a 2008 study by Anderson and colleagues that aimed to establish a model system for examining biofilm formation in the CF lung, which noted that supplementation of CFBE41o^−^ *P. aeruginosa* cocultures with 0.4% arginine reduced bacterial virulence (22), a trait that is associated with the biofilm lifestyle. The same research group later showed that growth on arginine-containing medium leads to increased levels of the probiofilm signaling molecule cyclic di-GMP in *P. aeruginosa* (14). Recently, it was also demonstrated that administration of l-arginine to burn wounds decreased *P. aeruginosa* motility and dissemination (23). Together, these studies support the concept that extracellular arginine may favor the less virulent, but more recalcitrant, biofilm mode of growth at the cell surface.

Alternatively or in addition to extracellular-arginine depletion, SLC6A14 transporter function may exert an anti-infective effect by providing intracellular arginine as a substrate for the generation of the membrane-permeating antibacterial agent nitric oxide (NO). NO is known to be a key component of the host defense against infectious disease, and NO deficiency is well recognized in the CF population (for a review, see reference 24). Hence, SLC6A14 may function to provide an intracellular substrate for NO as an important part of the host defense in CF-affected airway tissues. Importantly, we were able to show that conditioned medium from SLC6A14-inhibited (i.e., α-MT-treated) primary bronchial cultures also affected the attachment of *P. aeruginosa* to abiotic surfaces under host cell-free conditions, an observation that corroborates that the effect noted in our study can be, at least in part, attributed to higher levels of extracellular arginine proximal to the epithelial cell surface and not simply reduced NO-dependent bacterial killing.

Importantly, and as expected from clinical observation, the attachment of exogenously applied *P. aeruginosa* was significantly greater in primary cultures of CF bronchial epithelium cells than in cultures generated from non-CF individuals (Fig. 5). The basis for this increased propensity for *P. aeruginosa* attachment in CF cultures was not specifically interrogated in the present study, as it has been well described previously (22, 25–27). Enhanced *P. aeruginosa* attachment to CF epithelium may relate to the reduced pH of the ASL, as acidification is known to increase *P. aeruginosa* growth and enhance attachment (28). Alternatively, the well-documented high viscosity of the mucus layer overlying CF airway cultures (29–31) may also contribute to the increased *P. aeruginosa* attachment that we observed in the present study. Further, although the primary cultures derived from CF lung transplants are maintained in the presence of a cocktail of antibiotics, we cannot exclude potential contamination with viruses. For example, Bomberger and colleagues (32) showed recently that coinfection with respiratory syncytial virus (RSV) led to dysregulation of the normal host mechanisms for the regulation of *P. aeruginosa* infection. In that study, the authors showed that RSV infection impaired the host-mediated sequestration of iron, a factor essential for biofilm formation (32). Surprisingly, we did not observe higher levels of *SLC6A14* expression in airway cultures derived from CF transplant patients than in those from healthy non-CF individuals. As the lungs of these CF patients were chronically infected prior to transplantation, we would have predicted, on the basis of our studies of HBE cultures exposed to flagellin, that *SLC6A14* expression would be enhanced (Fig. 3). However, as these cultures were aggressively treated with antibiotics to maintain culture viability, they may not be appropriate for modeling of the infection status of the CF lung *in vivo*.

In contrast to the studies using non-CF primary (i.e., HBE) cell cultures, inhibition of SLC6A14 in cultures derived from CF airway tissues did not increase the level of *P. aeruginosa* attachment. We interpret this finding to suggest that the protective effect of SLC6A14 function has been overwhelmed in CF bronchial cell cultures because of the greater bacterial burden in this cell type than in HBE cells (for reasons discussed above) or because of attributes specific to the primary cell tissues. Specifically, these cultures were derived from individuals with end-stage lung disease who had been exposed to chronic infection and tissue inflammation. Given the association of *SLC6A14* with the age at first infection in association studies, we propose that it could exert a protective effect in tissues from younger subjects who have not seen significant damage and a multitude of therapeutic interventions. Furthermore, if some of the patient samples used in this study contained polymorphisms in *SLC6A14*, as identified by GWAS (10, 11), the SLC6A14 in these samples may be insensitive to functional inhibition by α-MT; thus, a further increase in the attachment of *P. aeruginosa* over and above the basal (i.e., untreated) level would not be seen. In an attempt to address this discrepancy, we also completed coculture infections with monolayers generated from a cell line overexpressing F508del-CFTR (CFBE41o^−^) that did show a modest but significant increase in bacterial attachment when treated with the SLC6A14 inhibitor, consistent with the hypothesis that SLC6A14 function reduces bacterial adherence to CF epithelium. In future studies, we will test our prediction regarding the protective effect of SLC6A14 in an alternative model of patient-specific tissue, namely, nasal epithelial cultures, as this model is expected to exhibit fewer consequences of chronic disease than the transplanted lung.

Importantly, there are also multiple bacterium-related factors that may impact the protective role of SLC6A14 in the progression of *P. aeruginosa* infection. In the present work, we used a strain of *P. aeruginosa* that is a prototrophic flagellated lab strain with good biofilm-forming (i.e., surface attachment) capabilities (33). It is well documented that a variety of *P. aeruginosa* isolates, with a range of phenotypes, may be present during the course of a CF patient’s lifetime (for a review, see reference 34). Many clinical strains of *P. aeruginosa* isolated from chronic infections show decreased flagellin expression. In a study by Mahenthiralingam et al., 39% of 1,030 *P. aeruginosa* clinical isolates collected were nonmotile, although most early-CF and environmental isolates had both flagella and pili (35). Additionally, some bacteria are auxotrophic (i.e., unable to synthesize certain amino acids) and would require exogenous sources of specific amino acids for metabolism. There is evidence that the prevalence of auxotrophs may increase during pulmonary exacerbations (36, 37). Specifically, Taylor et al. showed that 86% (32/37) of the *P. aeruginosa* bacteria isolated from infected adult patients in their study were auxotrophs and that this phenotype was associated with more severe disease, although the specific amino acid requirements were not investigated (36). Other studies have shown that auxotrophic variants develop from the wild-type strain of *P. aeruginosa* over time in CF patients and that arginine deficiency is among the three most prevalent auxotrophies, along with those of methionine and leucine (38). These observations suggest that SLC6A14 function may be most important during early exposure and/or infection, when bacteria are expressing flagella, and suggest that control of extracellular levels of arginine by SLC6A14 may have variable impacts depending on the nutritional requirements of the bacterial strains present over time.

**FIG 8:**
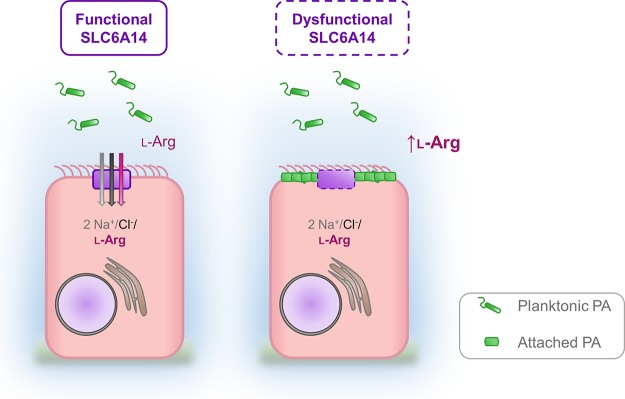
Extraction of l-arginine from ASL by SLC6A14 contributes to host defense by reducing the attachment of *P. aeruginosa* (PA) to the apical membrane of airway epithelial cells. The amino acid transporter SLC6A14 (solid purple box) mediates the uptake of l-arginine into ciliated epithelial cells in the lung. When SLC6A14 is dysfunctional (dashed purple box) because of genetic variation (e.g., polymorphisms), abrogated gene expression, or transporter inhibition (e.g., by α-MT), l-arginine accumulates in the ASL and stimulates the attachment of *P. aeruginosa* to the surface of epithelial cells.

In summary, we show in both mice and humans that the functional expression of *SLC6A14* acts to mitigate the initial stages of colonization of the airways by *P. aeruginosa* and that it does so by transporting an attachment-promoting metabolite for the bacterium out of the host ASL (Fig. 8). Furthermore, the expression of this gene is regulated by the presence of FLA-PA, arguing that it constitutes an important component of host immunity. Hence, SLC6A14 may modify CF lung disease at the age at first exacerbation in part because of its role in host defense.

## MATERIALS AND METHODS

### Reagents and buffers.

Unless otherwise stated, all reagents were purchased from Sigma-Aldrich, St. Louis, MO. The HEPES buffer used for all assays contained 25 mM HEPES, 140 mM sodium chloride, 5.4 mM potassium chloride, 1.8 mM calcium chloride, 0.8 mM magnesium sulfate, and 5 mM glucose.

### Animals.

The *Slc6a14*^−/y^ mouse line was created under the National Institutes of Health Knockout Mouse Project (KOMP) and obtained from the KOMP repository (http://www.komp.org). The genetic mutation was created by *Cre* and FLP recombination in a C57BL/6N background.

### Epithelial cell culture.

The human bronchial adenocarcinoma cell line Calu-3 was purchased from the American Type Culture Collection (Manassas, VA) and maintained with Eagle’s minimum essential medium (EMEM) with 10% fetal bovine serum and 1% penicillin-streptomycin. Calu-3 cells were grown as confluent monolayers attached to plastic tissue culture-treated plates, and assays were performed 1 to 2 days postconfluence. The CFBE cell line, overexpressing F508del CFTR (CFBE41o-ΔF508), was received from Dieter Gruenert (University of California, San Francisco) and maintained with EMEM with 10% fetal bovine serum, 1% penicillin-streptomycin, and 300 μg/ml hygromycin B. CFBE41ο-ΔF08 cells were grown as a confluent monolayer on collagen IV-coated polyester membrane inserts (24-well plates, 0.4 µm pore size) (Transwell inserts; Corning, Corning, NY), and assays were performed 18 days postseeding. Primary airway cultures were generated from posttransplant bronchial tissue from healthy donors (HBE cells) and CF patients (CFBE cells) (NIH Iowa Culture Facility). Cells were also plated on collagen IV-coated polyester membrane inserts (12-well plates, 0.4 µm pore size). Seeding on these inserts allows for cell polarization and gives access to both the apical and basolateral compartments, allowing cells to be grown at the air-liquid interface. Postseeding, medium was not present in the apical compartment and the medium in the basolateral compartment was changed every 2 to 3 days, maintaining cultures at the air-liquid interface. Primary airway culture medium consisted of a 1:1 mixture of Dulbecco’s modified Eagle’s medium (DMEM) and Ham’s F-12 medium supplemented with 2% Ultroser G Serum Substitute (PALL France SAS, Saint Germain-en-Laye, France), 50 U/ml penicillin, 50 μg/ml streptomycin, 50 μg/ml gentamicin, 2 μg/ml fluconazole, and 1.25 μg/ml amphotericin B.

### Quantitative RT-PCR.

Cells were lysed, and RNA extraction was performed in accordance with the protocol outlined by the RNeasy Plus minikit (Qiagen, Hilden, Germany). Following cell lysis and extraction, the RNA concentration was measured with a NanoDrop 2000 (ThermoFisher Scientific, Waltham, MA, USA). Samples were used with concentrations of >100 ng/μl, with a 260/280 ratio of 2.0 to 2.2. The iScript cDNA synthesis kit (Bio-Rad, Hercules, CA), containing reverse transcriptase enzyme, was used to make 1 μg of cDNA for each sample (control samples were RNA incubated with water in place of reverse transcriptase). Quantitative RT-PCR was performed with the EvaGreen fluorophore (SsoFast EvaGreen Supermix with Low Rox; Bio-Rad) in a 96-well plate format with a CFX96 Touch RT-PCR detection system (Bio-Rad). The sequences of the primers used are listed in Table S1. Fold changes in transcript levels were calculated by the 2^−ΔΔ*CT*^ method (39).

### Prediction of *SLC6A14* binding sites and STAT inhibition experiments.

The HaploReg v2.0 database (Broad Institute, MIT and Harvard) (40) was used to extract variants that disrupt STAT motifs or have STAT transcription factor binding sites detected through chromatin immunoprecipitation-DNA sequencing in ENCODE cells around chrX:115.35 to 115.65 Mb (hg19), a region selected to include intergenic genomic variation 5′ of the *SLC6A14* gene, where an association with CF lung disease has been reported (9). To test the effect of STAT inhibition, Calu-3 cell monolayers were treated with dimethyl sulfoxide (DMSO; vehicle control), FLA-PA (1 µg/ml; InvivoGen, San Diego, CA, USA), nifuroxazide (1 µM; Selleck Chemicals, Houston, TX), or a combination of FLA-PA and nifuroxazide (at the previously stated concentrations) for 2 h. Cells were lysed, and RNA was extracted for quantitative RT-PCR as described above.

### Quantification of l-arginine uptake by epithelial monolayers.

l-[2,3-^3^H]arginine was used to monitor the intracellular uptake of arginine from ASL by epithelial cell monolayers grown on plastic (Calu-3) or by segments of mouse trachea. For Calu-3 cells, cell monolayers were washed and then incubated for 30 min in HEPES buffer at 37°C to remove cell culture medium and reduce the intracellular pool of amino acids present. Cells were then treated with HEPES buffer supplemented with 1 µCi/ml l-[2,3-^3^H]arginine (specific activity, 54.6 Ci/mmol) and 100 µM unlabeled l-arginine in the presence or absence of the pharmacological blocker α-MT (at a final concentration of 2 mM) for 15 min. Control samples consisted of HEPES buffer supplemented with 1 µCi/ml l-[2,3-^3^H]arginine and 10 mM unlabeled l-arginine to outcompete the uptake of the radiolabeled amino acid. Following this, cells were washed three times with ice-cold HEPES buffer supplemented with 10 mM arginine and lysed with 0.5 M sodium hydroxide for 10 to 15 min while shaking on ice. A 12.5 µl volume of each lysate was added to 2 ml of EcoScint A Scintillation Fluid (Diamed, Cressier, Switzerland), and counts were read with a Beckman scintillation counter (LS-6000IC) that gives a readout in counts per minute. The total amount of protein in each sample was determined with a protein assay based on the method of Bradford (Bio-Rad, Hercules, CA).

For assays with murine tissue, tracheal segments from wild-type (*Slc6a14*^+/y^) and *Slc6a14*^−/y^ (knockout) C57BL/6N mice were injected with l-[2,3-^3^H]arginine-containing fluid in accordance with the protocol described above with 20 mM unlabeled l-arginine as the control treatment. Following uptake, loops were excised open and washed with ice-cold HEPES buffer and the entire tissue segment was lysed with 0.5 M sodium hydroxide for 1 h on ice. Samples were centrifuged at 18,000 × *g*, supernatants were collected, and counts per minute were determined as described above. The counts per minute obtained from each reading were taken as a ratio of whole-tissue lysate from each sample, expressed as follows: (counts per minute of l-[2,3-^3^H]arginine/mg of protein/ml)_treatment_ − (counts per minute of l-[2,3-^3^H]arginine/mg of protein/ml)_background_. The background number of counts per minute (HEPES buffer only) is the amount of l-[2,3-^3^H]arginine in the presence of 10 to 20 mM (unlabeled) l-arginine (maximal uptake competition by either nonspecific transport or diffusion into the cell) and is the background control for the assay.

### Quantification of transporter substrates in ASL.

ASL was collected from the apical side of primary epithelial cell monolayers grown on Transwell inserts following a 6-h exposure to buffer (negative control) or FLA-PA (1 μg/ml), followed by the addition of DMSO (vehicle control), l-arginine (80 to 110 μM), and/or α-MT (2 mM) for 1 h. Amino acid content was determined via HPLC with a reverse-phase C_18_ column.

### Bacteria.

All experiments were done with *P. aeruginosa* PAO1 carrying plasmid pMF230, which allows for the constitutive expression of enhanced GFP (eGFP) (41). Bacteria were maintained on Pseudomonas Isolation Agar (PIA; Becton, Dickinson and Company, Franklin Lakes, NJ, USA), grown in Trypticase soy broth (Becton, Dickinson and Company), and suspended in HEPES buffer (described above) with or without 20 mM l-arginine (pH 7.4) prior to growth assays or coculture experiments. All medium types were supplemented with 300 µg/ml carbenicillin to maintain eGFP expression.

### Biofilm/planktonic growth assays.

Microtiter plate biofilm assays were performed on the basis of the protocol of O’Toole (42). Briefly, overnight cultures of *P. aeruginosa* PAO1/pMF230 were adjusted to an optical density at 600 nm (OD_600_) of 1.0 in HEPES buffer. Five microliters of the adjusted culture was added to 195 μl of sterile HEPES buffer (with or without l-arginine and supplemented with carbenicillin) or epithelial-cell-conditioned HEPES buffer and incubated at 37°C under 5% CO_2_ in the wells of a 96-well polystyrene plate for 24 h. At the end of the assay, the unattached (i.e., planktonic) bacteria were removed and placed in a sterile microtiter dish and the cell OD_600_ was measured with a plate reader. Biofilm biomass was quantified by measuring the absorbance at 590 nm (*A*_590_) of solubilized crystal violet. For bronchial epithelial cell conditioning, HEPES buffer was collected from the apical side of primary cell cultures that had been incubated for 8 h with or without α-MT (2 mM) at 37°C under 5% CO_2_.

### Coculture infection assays.

Twenty-four hours prior to infection, the maintenance cell culture medium in the basal compartment of the Transwell insert was exchanged for FluoroBrite DMEM (ThermoFisher Scientific, Waltham, MA, USA) to reduce the background fluorescence during confocal imaging procedures and to remove antibiotics that could inhibit bacterial growth. At 0 h, *P. aeruginosa* PAO1/pMF230 suspended in HEPES buffer (containing 20 mM l-arginine with or without 2 mM α-MT) was added to the apical side of the Transwell at a multiplicity of infection of 2. The cocultures were then incubated at 37°C under 5% CO_2_ for 8 h (primary cell monolayers) or 6 h (cell line monolayers). Following infection, the HEPES buffer was removed for planktonic cell counts (if applicable) and the monolayers were washed with sterile phosphate-buffered saline (PBS). For attached bacterial counts, the membrane was carefully cut from the Transwell insert with a sterile blade and placed in cell dissociation reagent (TrypLE Express; ThermoFisher Scientific) for 15 min at 37°C. Dissociated cells and attached bacteria were vortexed (10 s), diluted in PBS, and plated on PIA (Becton, Dickinson and Company, Franklin Lakes, NJ, USA) for quantification of the number of CFU. Primary cell monolayers were monitored for damage by light microscopy, and TER measurements were taken at 0 and 8 h (Millicell ERS volt-ohm meter; EMD Millipore, Billerica, MA). Cell line monolayers were monitored for damage by light microscopy, and TER measurements were taken at 0 h (minimum for use, 500 Ω).

### Confocal laser scanning microscopy.

Following 8 h of infection with *P. aeruginosa* PAO1/pMF230, primary human epithelial cell monolayers were washed with PBS, carefully removed from the Transwell inserts with a sterile blade, mounted on a glass slide, and covered with a glass coverslip. Samples were viewed on an upright Leica DM6000B microscope connected to a Leica TSC SP5 system at the Molecular and Cellular Imaging Facility, University of Guelph. Images were acquired with a Leica digital camera (DFC350FX) and Leica LAS AF imaging software v1.6.0 (Leica, Wetzlar, Germany).

### Murine explant infection assays.

Two-hour tissue explant infections were performed on the basis of the methods of Harrison et al. (19) with tissue collected from 60-day-old male C57BL/6N wild-type (*Slc6a14*^+/y^) or *Slc6a14*^−/y^ (knockout) mice. The tracheal segments were dissected from the base of the larynx to the tracheal bifurcation (prior to the carina), cut into two sections, and placed in a microtube containing 1.0 ml of *P. aeruginosa* PAO1/pMF230 diluted in HEPES buffer (with 20 mM l-arginine) at an OD_600_ of 1.0. The left and right lungs of each mouse were cut at the primary bronchi and placed in separate microtubes containing bacteria as described above. The samples were incubated at 37°C under 5% CO_2_ for 2 h and then washed twice in sterile PBS (vortexed for 10 s each time) to remove nonadherent bacteria. Lung tissues were homogenized and plated on PIA for quantification of CFU. For visualization by scanning electron microscopy, mouse tracheas were washed with PBS and then placed in fixative (4% paraformaldehyde and 2.5% glutaraldehyde in 0.1 M sodium cacodylate buffer) overnight at 4°C. The following day, the samples were washed in 0.1 M sodium cacodylate buffer, incubated with 2% osmium tetroxide for 30 min, and then dehydrated in a series of ethanol washes (50, 70, 80, 90, and 100% for 10 min each). Whole tracheas were critical point dried (DCP-1; Denton Vacuums, Moorestown, NJ), dissected along the open side of the C-shaped rings of hyaline cartilage, flat mounted onto specimen stubs, and then coated with gold (Desk V deposition system; Denton Vacuums). All images were acquired on a Quanta FEG 250 scanning electron microscope (FEI, Hillsboro, OR) in high-vacuum mode (10,000 kV) at ×5,000 magnification with a spot size of 2 and a working distance of 10 mm.

### Statistical analysis.

Statistical analysis was completed with Prism software (GraphPad Software, Inc., La Jolla, CA). Data with single comparisons were assessed with unpaired or paired (where applicable) *t* tests with α = 0.05. Two-tailed tests were used for all experiments, with the exception of the murine infection data. Nonparametric (Mann-Whitney) tests were used when the mean of the control group was equal to zero, and a Welch correction was applied when groups had significantly different variances, as indicated by an *F*-test *P* value of <0.05. Data with multiple comparisons were assessed with Tukey’s multiple-comparison tests with α = 0.05. Technical replicates (when indicted) were averaged prior to analysis so that all data points represent biological replicates.
