# Supplementary figures and images for: SLC6A14 Is a Genetic Modifier of Cystic Fibrosis That Regulates Pseudomonas aeruginosa Attachment to Human Bronchial Epithelial Cells
Source: mBio. 2017 Dec 19;8(6):e02073-17. doi: 10.1128/mBio.02073-17 (PMC5736915; doi:10.1128/mBio.02073-17)

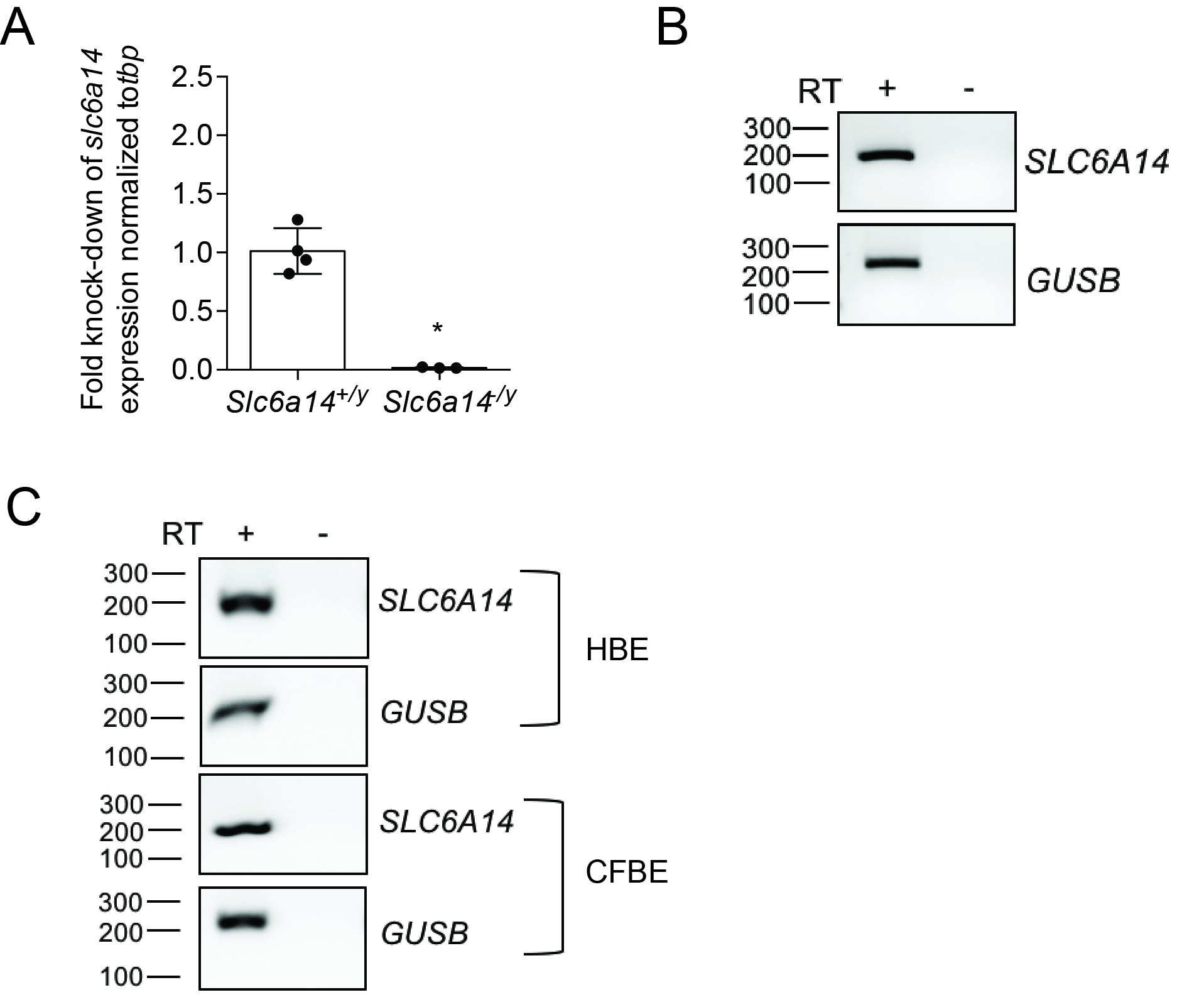

Supplement: FIG S1 [file mbo006173652sf1.tif]

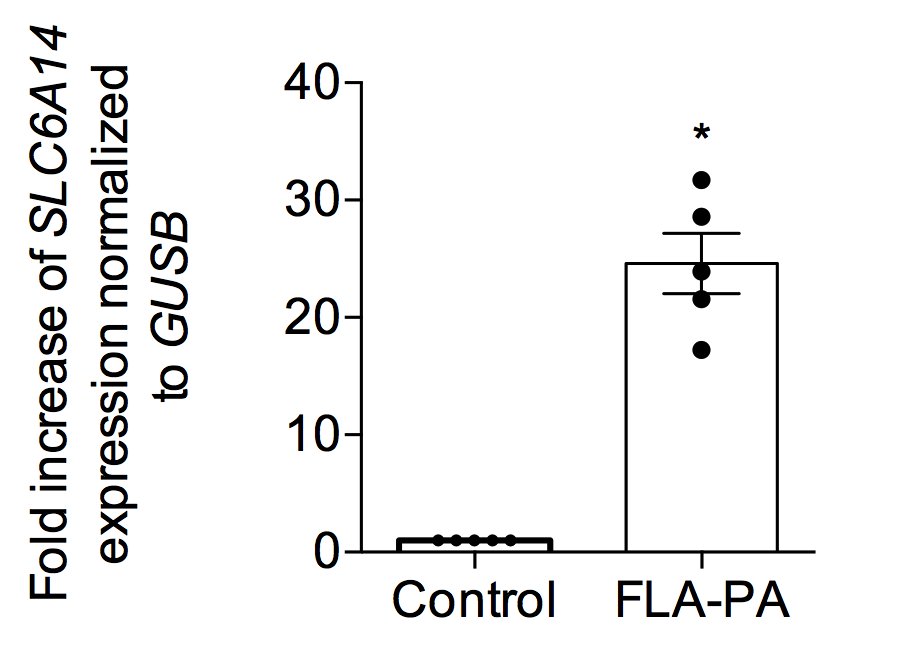

Supplement: FIG S2 [file mbo006173652sf2.tif]

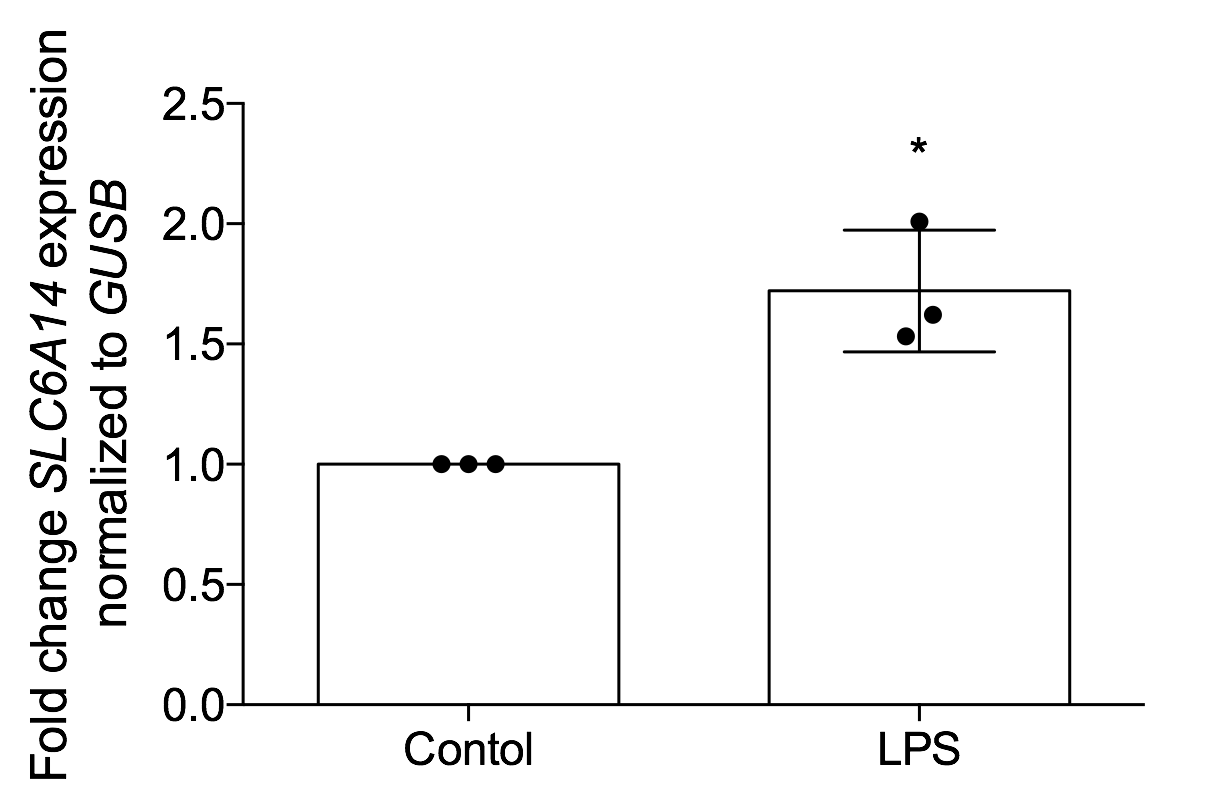

Supplement: FIG S3 [file mbo006173652sf3.tif]

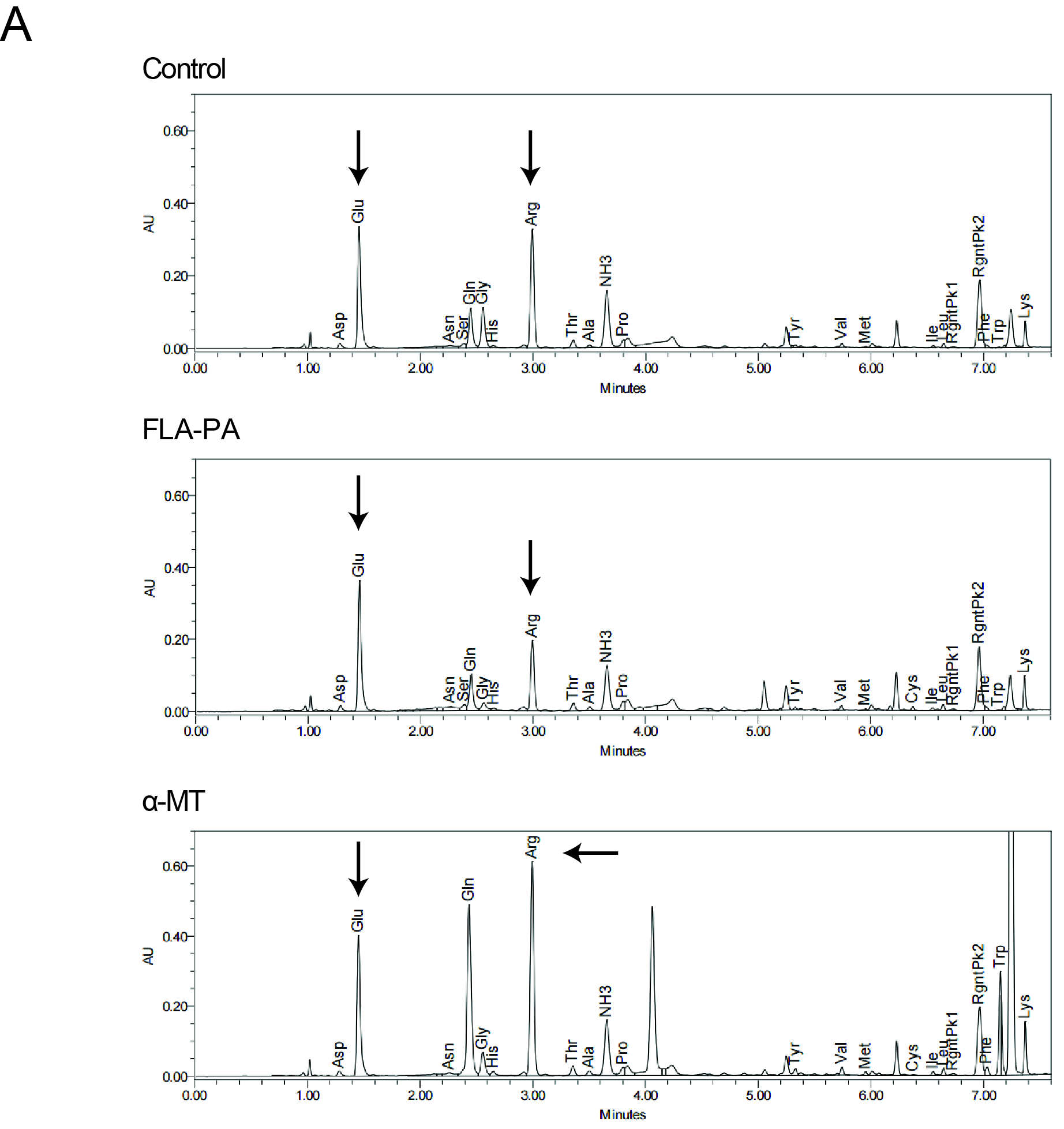

Supplement: FIG S4A [file mbo006173652sf4a.tif]

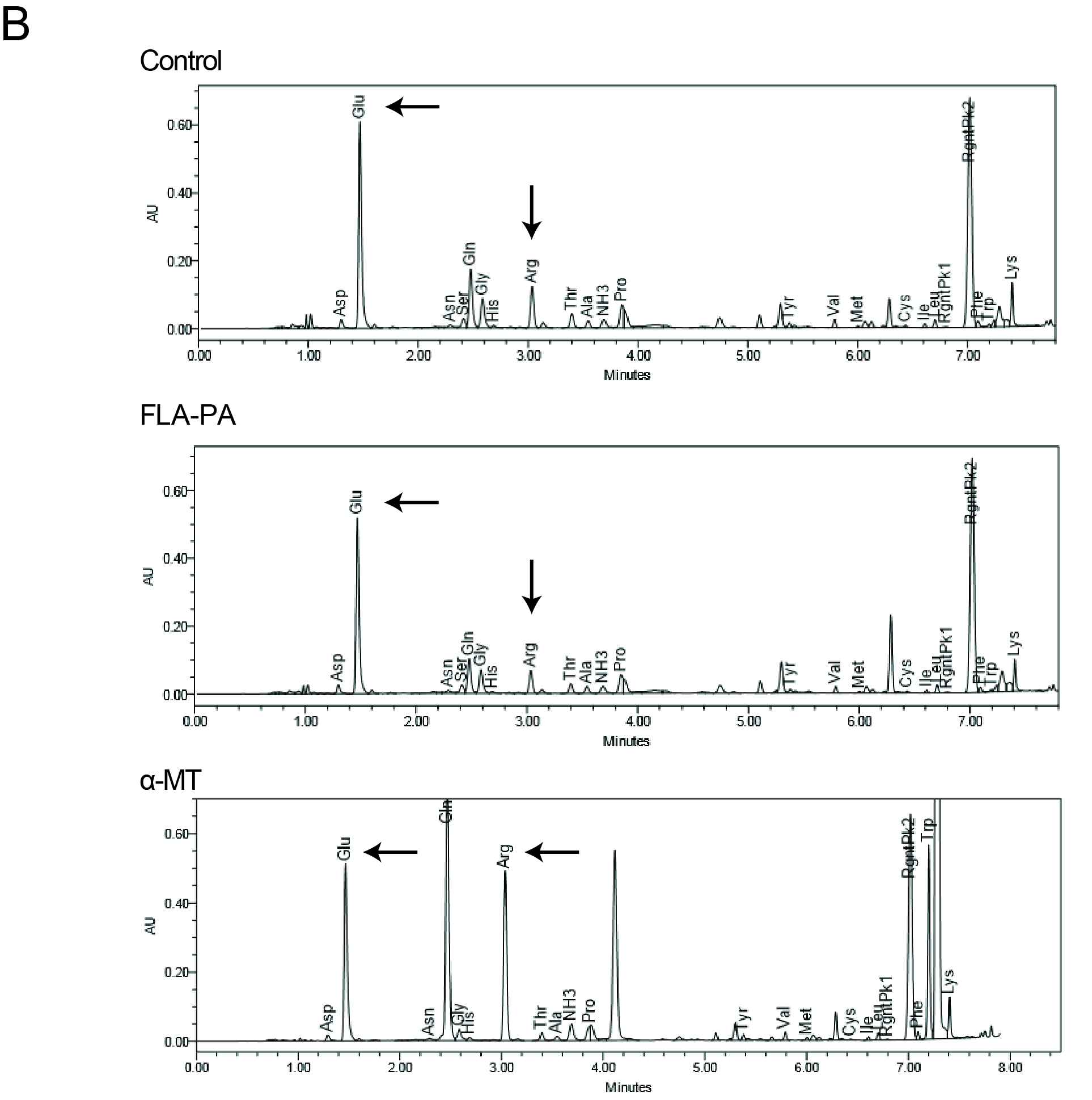

Supplement: FIG S4B [file mbo006173652sf4b.tif]

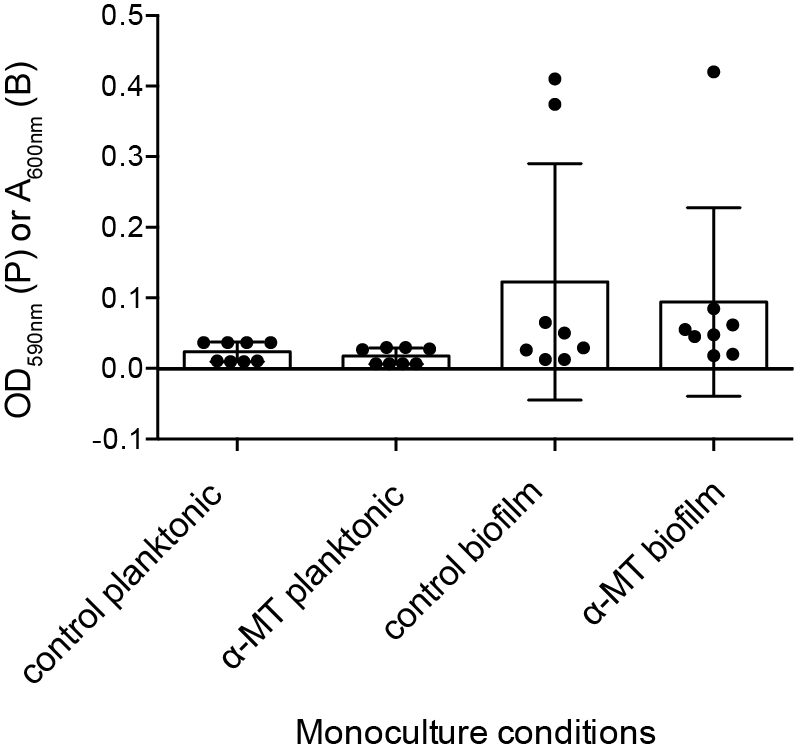

Supplement: FIG S5 [file mbo006173652sf5.tif]

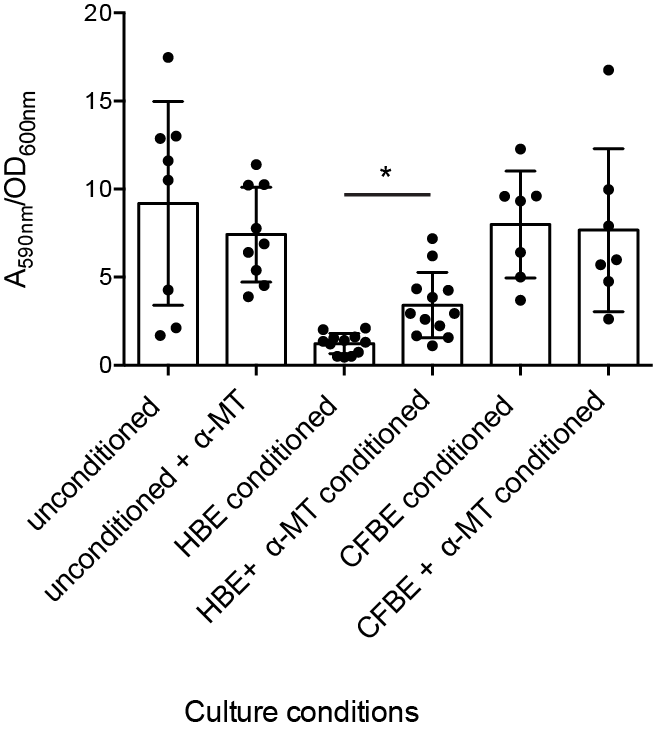

Supplement: FIG S6 [file mbo006173652sf6.tif]

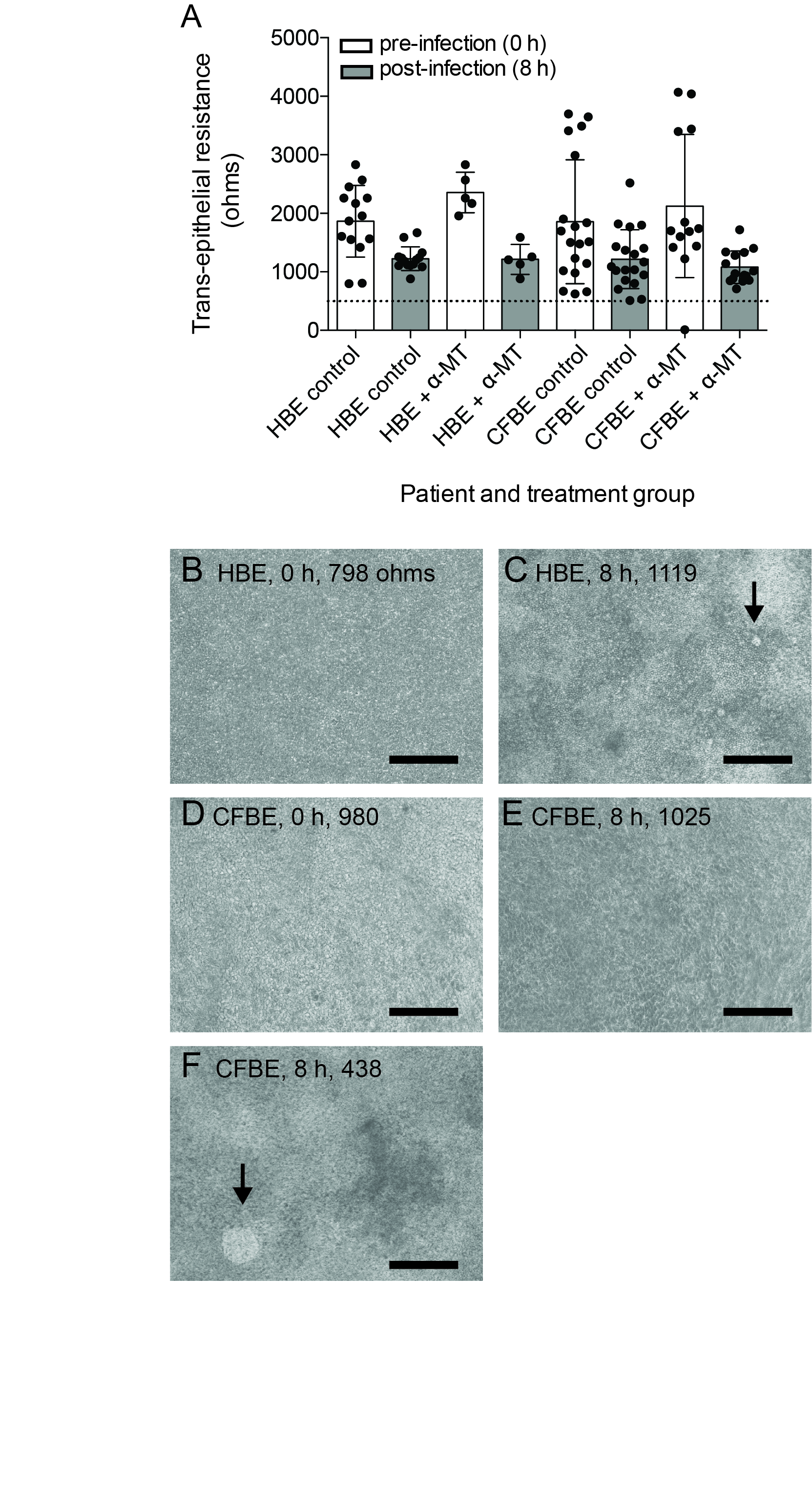

Supplement: FIG S7 [file mbo006173652sf7.tif]
