# Supplementary material for: SLC6A14 Is a Genetic Modifier of Cystic Fibrosis That Regulates Pseudomonas aeruginosa Attachment to Human Bronchial Epithelial Cells
Source: mBio. 2017 Dec 19;8(6):e02073-17. doi: 10.1128/mBio.02073-17 (PMC5736915; doi:10.1128/mBio.02073-17)
Supplement: TABLE S1 [file mbo006173652st1.docx]

**Table S1. Primer sequences used for quantitative real-time PCR assays.**

| **Gene** | **Species** | **Forward Primer (5’-3’)** | **Reverse Primer (5’-3’)** |
| --- | --- | --- | --- |
| *SLC6A14* | Human | TATGGCGCAATTCCATACCC | CCAGGTATGGACCCCAGTTA |
| *GUSB* | Human | CCCATTATTCAGAGCGAGTATG | CTCGTCGGTGACTGTTCAG |
| *Slc6a14* | Murine | GCTTGCTGGTTTGTCATCACTCC | TACACCAGCCAAGAGCAACTCC |
| *Tbp* | Murine | CAAACCCAGAATTGTTCTCCTT | ATGTGGTCTTCCTGAATCCCT |
