# Supplementary material for: SLC6A14 Is a Genetic Modifier of Cystic Fibrosis That Regulates Pseudomonas aeruginosa Attachment to Human Bronchial Epithelial Cells
Source: mBio. 2017 Dec 19;8(6):e02073-17. doi: 10.1128/mBio.02073-17 (PMC5736915; doi:10.1128/mBio.02073-17)
Supplement: TABLE S2 [file mbo006173652st2.docx]

**Table S2. Genotype table.** *CFTR* genotypes of each CFBE primary cell culture used for each of the indicated assays.

| **Quantitative RT-PCR** | **Collection of ASL** | **Co-culture** |
| --- | --- | --- |
| *F508del/R347H* | *F508del/F508del* | *F508del/F508del* |
| *F508del/F508del* | *F508del/c.2050dupA* | *F508del/c.2050dupA* |
| *F508del/F508del* | *F508del/M110K* | *F508del/M110K* |
| *F508del/F508del* | *F508del/F508del* | *F508del/F508del* |
| *F508del/2622+1G>A* | *D529G/V520F* | *D529G/V520F* |
|  | *F508del/F508del* | *F508del/F508del* |
|  |  | *F508del/M110K* |
|  |  | *F508del/F508del* |
|  |  | *F508del/F508del* |
|  |  | *F508del/F508del* |
|  |  | *F508del/F508del* |
|  |  | *F508del/2622+1G>A* |
|  |  | *F508del/F311del* |
